# Shared and nonshared agency for occupational goals with mothers, fathers, VIPs, and romantic partners during young adulthood

**DOI:** 10.3389/fpsyg.2022.902288

**Published:** 2022-11-18

**Authors:** Esther S. Chang, Jacob Shane, Brandilynn Villarreal, Jutta Heckhausen

**Affiliations:** ^1^Social and Behavioral Sciences, Soka University of America, Aliso Viejo, CA, United States; ^2^Department of Psychology, Brooklyn College (CUNY), Brooklyn, NY, United States; ^3^Department of Psychology, Cal Poly Humboldt, Arcata, CA, United States; ^4^Department of Psychological Science, University of California, Irvine (UCI), Irvine, CA, United States

**Keywords:** occupational development, family influence, romantic partner influence, mentoring, career development

## Abstract

This study applied a framework of shared and nonshared agency to investigate how social partners can help and hinder young adults’ career development. We also considered the extent to which motivational control could be promoted or burdened when young people seek help and encouragement from others in their careers. Based on the importance of shared agency in life goal pursuit, it was hypothesized that shared agency (i.e., perceived support and collaboration) with mothers, fathers, important adults, and romantic partners would have direct and positive associations with young adults’ career satisfaction and exploration and positive indirect associations on career development *via* motivational control. We further hypothesized that nonshared agency (i.e., directing and uninvolvement) would have direct and negative effects on career satisfaction and exploration and negative indirect effects on career development *via* motivational strategies. Results indicated that relationships can facilitate career development but differently depending upon relationship type. We found that support and directing from mothers and VIPs had positive associations with outcomes *via* individual motivational control whereas a total effect of collaboration with fathers and romantic partners were associated with outcomes without an indirect effect *via* motivational control. These findings are discussed within the context of previous socialization research and theory.

## Introduction

Young adults must prepare for and establish their careers within an increasingly globalized society characterized by rapidly changing social norms and technological innovations (Buchholz et al., 2009; Nagy et al., 2019). Although globalization may have expanded the range of possible occupations for young people, the risks include increased ambiguity and complexity of the labor market and planning one’s long-term career prospects (Shanahan et al., 2002; Klug et al., 2019; Schoon and Heckhausen, 2019; Shane and Heckhausen, 2019). The pathway to work for many young adults is increasingly difficult. To overcome the challenges of career development during young adulthood, individuals must be highly motivated and invested in their occupational goals, and receive guidance, information, and support from others to select and pursue these goals successfully (Higgins and Kram, 2001; Buhl et al., 2018).

The current study expands upon approaches from vocational and counseling psychology, which emphasize that employment is inherently social (e.g., Blustein, 2011; Lent and Brown, 2013). For example, Social Cognitive Career Theory (Lent et al., 1994), the Relational Theory of Working (Blustein, 2011), and the Developmental-Contextual Approach (Vondracek and Porfeli, 2008) have proposed that the quality of one’s social context is related to occupation-related expectations, feelings of self-efficacy, and levels of satisfaction. While social relationships are increasingly identified as important to career development, more research is needed to better understand how specific members in one’s social network help or hinder occupational choice and goal pursuit during the transition to adulthood. This study synthesizes previous approaches and contributes to the field by offering a framework of shared and nonshared agency that highlights how *social relationships* can act as a motivational unit in the process of career development.

### Social support for young adult career development

Previous research has shown that there are a variety of people close to a young adult who can be influential in career development. Yet, most of this research examines one social partner at a time and/or one type of involvement. It is well documented, for example, that social support is positively associated with early career development, particularly when the source of support originates from a network of family members (Fouad et al., 2010; Cotton et al., 2011; Metheny and McWhirter, 2013), an important nonparental adult (Garcia et al., 2015; Michaeli et al., 2018), or a romantic partner (Kvitkovičová et al., 2017; Buhl et al., 2018). It is also increasingly recognized that relationship partners can do more than merely support young adults’ career development. One can be a role model, encourage the young adult to act, and/or actively open new occupational pathways for the person (Higgins and Kram, 2001; Ginevra et al., 2018). At the same time, social partners can be uninvolved, forceful, and/or critical of young adults’ career choices and pursuit strategies (Phillips et al., 2001). The current study seeks to address an important next step in research on career development, which is to examine a variety of roles that different types of social partners can play in young adults’ career development.

Parents have long been identified as important sources of support for young adults’ career development (Kracke, 1997; Dietrich and Kracke, 2009), with mothers and fathers each influencing their children (Tynkkynen et al., 2010; Powers and Myers, 2017; Kantamneni et al., 2018; Michaeli et al., 2018). When studies separate out and compare the relative influence of support from mothers versus fathers, perceived maternal support has been found to be relatively more important to one’s career development (Powers and Myers, 2017; Kantamneni et al., 2018; Michaeli et al., 2018). Reasons for the importance of mothers, relative to fathers, are not well understood in the literature on career development but are consistent with literature on the socialization of children adolescents in general (e.g., Laible and Carlo, 2004). Since some studies suggest that young adults may seek more active forms of parental support regarding career choice, such as talking about the pros and cons of a job and discussing which occupations or jobs might be the best fit for them (e.g., Buhl et al., 2018), it is possible that mothers may be more likely to have these detailed conversations based on their prior caretaking history with their child. However, providing active forms of support for a young adult is not without risks. One must balance joint decision-making with the young adult’s need for autonomy, to avoid being perceived as interfering or controlling (Dietrich and Kracke, 2009). Moreover, active parental involvement may reflect parental expectations and values that serve to bind the family together (Young et al., 2001; Fouad et al., 2010). This may lead young adults to feel parental pressure as well as a sense of family obligation, which can either motivate individuals to strive harder or to disengage from their goals altogether. Thus, if goals are not congruent between parents and young adults, difficulties in young adults’ motivational regulation and career development can be expected.

Parents are not young adults’ sole source of career advice and support. Nonparental adults, such as older siblings, grandparents, aunts, uncles, coaches, professors, or other mentors can also serve as critical sources of encouragement and support for the developing person’s career. Nonparental adults, whom young adults identify as very important in their lives (“VIPs”; Greenberger et al., 1998), are thought to provide unique sources of social capital after high school. For example, youth who reported a highly educated VIP attained higher levels of education after high school relative to peers with VIPs who had lower levels of education (Chang et al., 2010a). Research on career development has also found that VIPs more than parents may be providing young adults with career-specific support (Tynkkynen et al., 2010; Powers and Myers, 2017; Michaeli et al., 2018). VIPs may have a unique influence on career development because they can be sought out and selected by young people when the need arises, and because they are adults, VIPs can be useful as a conduit to the adult occupational world.

Finally, the transition from school-to-work often happens simultaneously with young adults’ development of romantic relationships (Branje et al., 2014). Therefore, romantic partners can become a powerful but overlooked influence on a young adults’ career development (Kvitkovičová et al., 2017; Buhl et al., 2018). As one envisions cohabitating with another person over the long-term, interdependence of occupational goal selection and long-term prospects may become more salient with a romantic partner. Among the handful of studies focusing on romantic partner support of young adults’ career goals, relationship closeness and shared goals are important facilitators of goal attainment (Kornblum et al., 2021). Fitzsimons et al. (2015) found that couples with shared occupational goals allocated greater resources to goal pursuit, which enhanced the likelihood of goal attainment. Much more research on romantic partner support can be seen for those in mid-career. These studies have found spousal support to have an important impact on career success and adaptability (Masterson and Hoobler, 2015).

In the above-referenced studies, the concept of social support is often treated as a context variable for the individual to either use or to be influenced. Our goal is to show that the influence of important others in youth’s occupational choices, motivation and developmental outcomes can go way beyond support, for better or worse.

### Shared and nonshared agency for occupational goals

The approach we apply in the current study is rooted in the academic socialization literature that seeks to understand how parents can best negotiate adolescent autonomy (e.g., Barber, 1996; Baumrind, 2005). The framework of shared and nonshared agency maps out how these recognized parenting practices can be more broadly applied to the processes involved in how the child and their major social partners are interfacing in shaping the child’s life goals (Heckhausen et al., 2010; Chang et al., 2010b). When both the young adult and their social partner(s) are aligned in goal engagement, there is *shared agency*; when one or both in the relationship are disengaged, there is *nonshared agency*.

When applied to career development, the concept of “shared agency” with another person is the idea that both individuals in a relationship are invested in the young adult’s career goals. In other words, both relational partners are understood to be goal engaged or actively involved in young adult’s career goals. As the young adult invests time and energy into attaining their career goals, the social partner is actively encouraging, guiding, and/or advising them. Previous research set in the academic context of college education has confirmed qualitatively different types of shared agency (Chang et al., 2010b). We draw on two such forms here. The *support* subtype of shared agency refers to the classic notion of others as a source of *support* and encouragement in the pursuit of a goal someone else chose. The *collaborate* subtype of shared agency is present when both partners attempt to work together on a goal they both chose and pursue together.

Nonshared agency is when one partner in the relationship is goal disengaged. When the young adult is less interested in developing their career plans, the other person can attempt to guide and/or encourage the young person to be interested (i.e., *directing*). When others ignore, neglect, or do not feel responsible for a young adult’s career progress, the young adult may pursue their career goals without their social partners’ involvement (i.e., *uninvolved*).

Prior cross-sectional studies on shared and nonshared agency have found that young people expect, seek, and benefit subjectively and objectively from others’ interest in their education when available (e.g., Chang, 2013; Kriegbaum et al., 2016). During the college years, shared agency helps young adults manage their higher educational goals by promoting individual self-regulation (Kriegbaum et al., 2016). Studies have found that when receiving parental support, older youth will typically report high levels of satisfaction with their educational goal progress (Chang et al., 2010b). While prior research on educational shared and nonshared agency helps inform expectations, occupational goals and their successful attainment are more ambiguous and diverse. Thus, strategies of occupational shared and nonshared agency may differ in function from the educational domain.

### Present study

The previously summarized research on career and vocational development suggests that parents, nonparental adults, and romantic partners can all play supportive roles for young people. We apply the shared and nonshared agency framework to better understand how these social partners influence young adults’ career development, beyond providing general social support. We examine how shared and nonshared agency with social partners (mothers, fathers, important nonparental adult, and romantic partners) can influence young adults’ motivation to engage or disengage with occupational goals, and their career development. Our analyses examine the total effects of each strategy (i.e., support, collaboration, directing, and uninvolved) on career development outcomes for each social partner, and separates these total effects into direct effects on career development outcomes and indirect effects between shared and nonshared agency strategies and career outcomes mediated *via* influences on goal engagement and goal disengagement. Based on the importance of shared agency in life goal pursuit, and previous research in the educational domain (e.g., Kriegbaum et al., 2016), it was hypothesized that shared agency support and collaboration would have direct and positive effects on young adults’ career development (satisfaction and exploration), and positive indirect effects on career development *via* motivational strategies. More specifically, shared agency is expected to be positively associated with goal engagement and negatively associated with goal disengagement; and in contrast, nonshared agency is expected to be negatively associated with goal engagement and positively associated with goal disengagement. We further hypothesized that nonshared agency patterns (i.e., directing and uninvolved) would have direct and negative effects on career development (i.e., satisfaction and exploration), and negative indirect effects on career development *via* motivational strategies.

## Materials and methods

### Participants and procedure

Data come from four samples of participants who completed an online survey (*N* = 2,102). One sample was recruited and assessed *via* Amazon Mechanical Turk (*n* = 303). These participants were compensated $8.50 for completing the survey. The second sample was recruited from the human subjects pool of a large public university in the western U.S. (*n* = 739). The third and fourth samples were recruited from the human subjects pool of a large public university in the northeastern U.S. (northeastern sample 1 *n* = 564; northeastern sample 2 *n* = 496).^1^ All samples from universities were compensated with course credit for completing the survey. IRBs from co-authors’ respective institutions approved the study. The final analyzed sample was restricted to young adult participants aged 18–29 who had complete data on the variables of interest. The sample was predominately female (66.11%), racially/ethnically diverse (Asian/Asian American 25.99%, Black/African American 8.15%, Hispanic/Latinx 19.16%, Native Hawaiian or other Pacific Islander 2.01%, White 33.28%, Multiracial/Multiethnic or an unlisted race/ethnicity 11.42%), and with an average age of 21.16 years (*SD* = 3.20). Participants only answered occupational shared agency questions for relationships they had, which resulted in sample sizes ranging from 815 to 1,598 participants across the models.

### Measures

#### Demographics

Participants answered questions about their ethnic background, gender, and age.

#### Occupational shared and nonshared agency

Measures of shared agency (*support* and *collaboration*) and nonshared agency (*directing* and *noninvolvement*) were adapted from previous measures of shared agency for educational goals with parents (Chang et al., 2010b) to indicate perceptions of shared and nonshared agency relating to occupational goals with multiple social partners (i.e., mothers, fathers, VIPs, and romantic partners).

Participants first indicated whether they had someone in their life who was or had been a mother, someone who was or had been a father, and someone in their life who they would consider their romantic partner. Participants were also asked whether they had someone who they considered an important nonparental adult (VIP), using the following instructions “Finally, many people your age have an important adult in their lives other than their parents—someone they feel they can count on, and who will be there for them. For example, is there a relative such as an aunt or a grandparent, a teacher, a coach, a counselor, a colleague, a friend, or someone else who is really important in your life?” Participants then answered the shared and nonshared agency items for each relationship they indicated they had (mother, father, romantic partner, and VIP).

Each shared and nonshared agency subscale contained five items. Participants responded to each item using a 6-point scale (1 = *Strongly disagree*; 6 = *Strongly agree*). All measures had high internal consistency (perceived support *α*s ranged from 0.85 for VIP to 0.88 for romantic partner; perceived collaboration *α*s ranged from = 0.84 for VIP to 0.88 for father). Item wordings are presented in Table 1.

**Table 1:** Occupational shared agency scale item wordings.

Support
My () supports me in preparing for my occupation
I turn to my () for comfort when I run into difficulties in pursuing my occupational goal
It is important that my () is very supportive of how I manage my occupational goal pursuit
My () cheers me up when I am having a hard time achieving my occupational goal
My () is there to answer any questions I have about how to reach my occupational goal
Collaboration
My () encourages me to seek information about occupations I am interested in
It helps me to get advice from my () in preparing for my future occupation
Me and my () are a team when it comes to trying to reach my occupational goals
I feel lost when my () is not there to help me reach my occupational goals
It is helpful when my () tells me about his/her occupational experiences
Directing
My () tries to push me in a certain direction in how I pursue my future occupation
My () tells me what I should be doing in order to reach my occupational goal
It is just easier to follow what my () tells me is best in how to achieve my occupational goal
My () makes me do what s/he thinks is best for my occupation
My () criticizes me if I am not doing what s/he thinks I should be doing in order to reach my occupational goal
Noninvolvement
My () does not think I have questions about how I should try to reach my desired occupation
My () provides no guidance to me about how I should reach my desired occupation
My () does not feel responsible for helping me achieve my occupational goals
My () does not ask me how I am doing in my occupational pursuits
I do not need any of my ()'s help to accomplish my occupational goals

Confirmatory factor analyses were also performed to establish the internal validity of the scale. For each relationship, the four-factor model (support, collaboration, directing, and noninvolvement) demonstrated good model fit (CFI = 0.90–0.92; RMSEA = 0.064–0.075; SRMR = 0.057–0.081) and fit the data better than the one-factor model, and any of the possible two- or three-factor models. The resulting measure demonstrated good internal consistency for perceived support (*α*s 0.85–0.88), collaboration (*α*s 0.84–0.88 for father), and directing (*α*s 0.80–0.81), and acceptable internal consistency for noninvolvement (*α*s 0.74–0.81 for father). Item wordings for the measurement are presented in Table 1.

#### Occupational motivational strategies

Two complementary types of occupational motivational strategies were measured: (1) *goal engagement* and (2) *goal disengagement* (Heckhausen et al., 1998; Shane and Heckhausen, 2016). The goal engagement scale contains six items (e.g., “I will work hard to have a good career”) and goal disengagement included four items (e.g., “If I am not successful in my career, I will know that it was not the right thing for me anyway”). Participants responded to each item on a six-point scale (1 = *Strongly disagree*; 6 = *Strongly agree*). Responses were averaged to create the composite career goal engagement measure (*α* = 0.84) and career goal disengagement measure (*α* = 0.67), with higher values reflecting greater goal engagement or goal disengagement, respectively.

#### Occupational outcomes

*Satisfaction* with progress toward occupational goals was measured using an eight-item occupation version of the educational satisfaction scale (Chang et al., 2010b). Participants indicated their level of agreement with each item (e.g., “I am very satisfied with my current progress toward reaching my occupational goal”) using a 6-point scale (1 = *Strongly disagree*; 6 = *Strongly agree*). Responses were averaged to create the composite occupational goal progress satisfaction measure (*α* = 0.90), with higher values reflecting greater satisfaction.

In addition, occupational *exploration* was measured using the six-item occupational exploration scale (Kracke, 1997). Participants indicated their level of agreement with each item (e.g., “I try to find out which occupations best fit my strengths and weaknesses”). Responses were averaged to create the composite occupational exploration measure (*α* = 0.88), with higher values reflecting greater occupational exploration.

## Results

**Figure 1:**
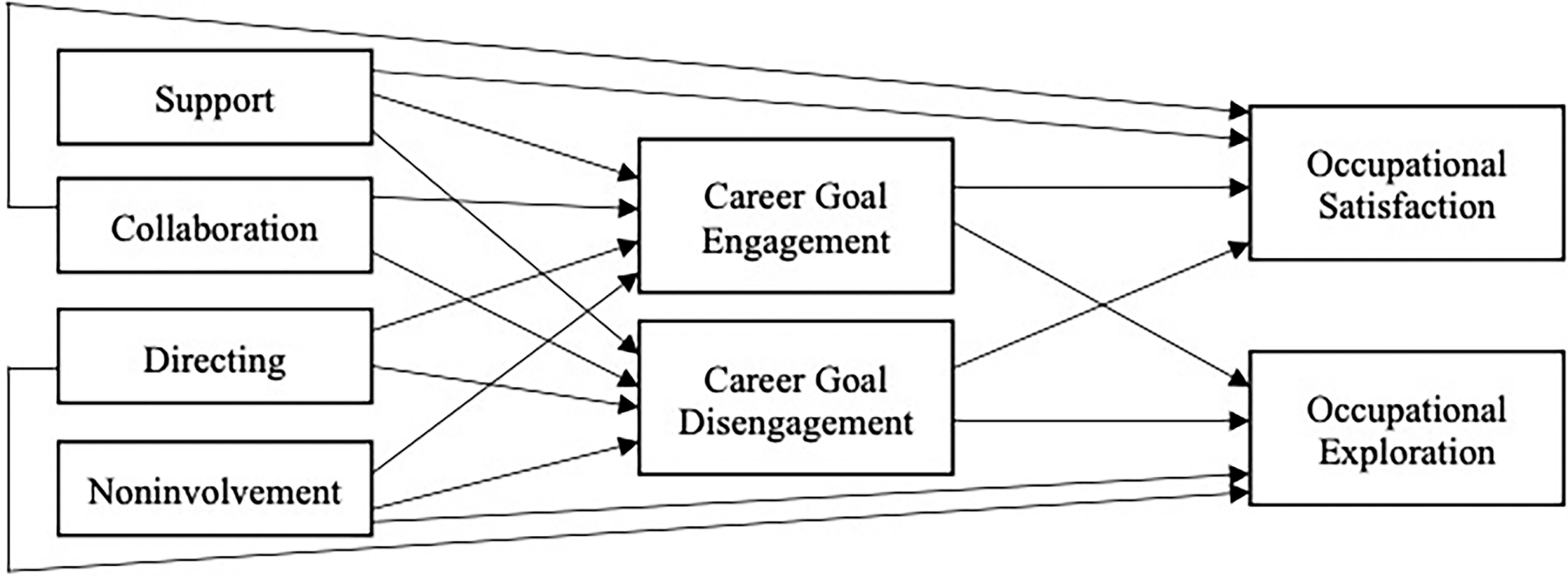
Mediation model depicting direct effects. Indirect effects are the effect of predictors (support, collaboration, directing, and noninvolvement) on outcomes (occupational satisfaction, and occupational exploration) *via* the mediators (career goal engagement, career goal disengagement). Total effects are the combination of direct and indirect effects. Covariances between predictors, between mediators, and between outcomes included in model but not depicted here. Age and gender included in model as covariates, but not depicted here.

Descriptive statistics and inter-item correlations for key study variables are provided in Table 2. Our primary analyses examined how perceptions of occupational shared (i.e., support and collaboration) and nonshared agency (i.e., directing and noninvolvement) with different social partners related to young adult’s career-related motivational strategies, occupational exploration, and satisfaction with occupational progress. Structural mediation models were setup for each social partner (i.e., mother, father, VIP, and romantic partner) wherein shared and nonshared agency predicted occupational exploration and satisfaction with occupational progress *via* career goal engagement and career goal disengagement (Figure 1). The model was assessed using the product of coefficients approach (Alwin and Hauser, 1975). This produced estimates of the total effects from shared and nonshared agency on occupational outcomes, as well as estimates of the direct effects from shared and nonshared agency on motivational strategies and occupational outcomes, and the indirect effects from shared and nonshared agency on occupational outcomes *via* career-related motivational strategies. Age and gender were controlled for in all analyses. Model fit statistics are not presented because the models were fully saturated. All analyses were performed in STATA 15. Results are presented in Tables 3–6 and summarized below.

**Table 2:** Descriptive statistics and interitem correlations for key study variables.

	*M* (SD)	1	2	3	4	5	6	7	8	9	10	11	12	13	14	15	16	17	18	19
**Mother**																				
1. Support	4.34 (1.36)																			
2. Collaboration	3.91 (1.83)	0.86^*^																		
3. Directing	2.90 (1.30)	0.17^*^	0.26^*^																	
4. Noninvolvement	2.41 (1.19)	−0.52^*^	−0.49^*^	0.05^*^																
**Father**																				
5. Support	3.85 (1.51)	0.61^*^	0.53^*^	0.11^*^	−0.35^*^															
6. Collaboration	3.56 (1.49)	0.56^*^	0.64*	0.18*	−0.34*	0.88*														
7. Directing	2.72 (1.28)	0.13^*^	0.18*	0.69*	0.06*	0.29*	0.37*													
8. Noninvolvement	2.63 (1.33)	−0.35^*^	−0.31^*^	0.05^*^	0.70^*^	−0.57^*^	−0.54^*^	−0.10^*^												
**VIP**																				
9. Support	4.35 (1.31)	0.40^*^	0.32^*^	−0.02	−0.21^*^	0.37^*^	0.30^*^	0.01	−0.13^*^											
10. Collaboration	3.99 (1.32)	0.35^*^	0.41^*^	0.07^*^	−0.18^*^	0.31^*^	0.36^*^	0.05	−0.09^*^	0.82^*^										
11. Directing	2.51 (1.24)	0.08^*^	0.15^*^	0.56^*^	0.15^*^	0.04	0.09^*^	0.48^*^	0.14^*^	0.26^*^	0.37^*^									
12. Noninvolvement	2.39 (1.12)	−0.24^*^	−0.22	0.15^*^	0.62^*^	−0.21^*^	−0.20^*^	0.15^*^	0.50^*^	−0.32^*^	−0.32^*^	0.08^*^								
**Romantic partner**																				
13. Support	4.58 (1.37)	0.26^*^	0.21^*^	−0.06	−0.12^*^	0.28^*^	0.23^*^	−0.004	−0.05	0.38^*^	0.32^*^	−0.02	−0.03							
14. Collaboration	4.01 (1.40)	0.28^*^	0.35^*^	0.03	−0.09^*^	0.27^*^	0.34^*^	0.06	−0.02	0.35^*^	0.42^*^	0.08^*^	−0.04	0.83^*^						
15. Directing	2.53 (1.26)	0.06	0.15^*^	0.52^*^	0.21^*^	0.12^*^	0.17^*^	0.50^*^	0.15^*^	0.04	0.11	0.56^*^	0.21^*^	0.27^*^	0.38^*^					
16. Noninvolvement	2.36 (1.15)	−0.18^*^	−0.14^*^	0.17^*^	0.56^*^	−0.17^*^	−0.14^*^	0.11^*^	0.47^*^	−0.18^*^	−0.16^*^	0.21^*^	0.61^*^	−0.16^*^	−0.18^*^	0.20^*^				
17. Career Goal Engagement	3.89 (1.12)	0.25^*^	0.19^*^	−0.07^*^	−0.26^*^	0.21^*^	0.18^*^	−0.05^*^	−0.18^*^	0.25^*^	0.22^*^	−0.06^*^	−0.24^*^	0.23^*^	0.18^*^	−0.08^*^	−0.21^*^			
18. Career Goal Disengagement	5.10 (0.69)	0.09^*^	0.10^*^	0.09^*^	−0.02	0.06^*^	0.06^*^	0.06^*^	−0.001	0.05^*^	0.09^*^	0.12^*^	0.04	0.05	0.09^*^	0.05	0.01	0.12^*^		
19. Occupational Satisfaction	2.82 (0.64)	0.25^*^	0.22^*^	−0.04	−0.20^*^	0.21^*^	0.20^*^	−0.04	−0.18^*^	0.18^*^	0.17^*^	0.02	−0.15^*^	0.13^*^	0.16^*^	−0.04	−0.13^*^	0.34^*^	0.01	
20. Occupational Exploration	4.74 (1.01)	0.19^*^	0.18^*^	0.02	−0.14^*^	0.17^*^	0.17^*^	0.01	−0.12^*^	0.19^*^	0.17^*^	0.03	−0.11^*^	0.18^*^	0.19^*^	−0.03	−0.12^*^	0.41^*^	0.11^*^	0.41^*^

The minimum reported value was 1 and the maximum reported value was 6 for all variables.

* *p* < 0.05.

**Table 3:** Direct, indirect, and total effects of shared and nonshared agency from mothers on occupational outcomes.

	Support		Collaboration		Directing		Noninvolvement		Disengagement		Engagement		Exploration		Satisfaction	
	*B(SE)*	*β*	*B(SE)*	*β*	*B(SE)*	*β*	*B(SE)*	*β*	*B(SE)*	*β*	*B(SE)*	*β*	*B(SE)*	*β*	*B(SE)*	*β*
**Direct Effects**																
Age	−0.03 (0.01)	−0.06^*^	−0.06 (0.01)	−0.13^***^	−0.07 (0.01)	−0.17^***^	0.03 (0.01)	0.09^***^	−0.01 (0.01)	−0.03	−0.0003 (0.01)	−0.001	−0.0001 (0.01)	−0.0002	0.01 (0.005)	0.03
Gender (1 = Female)	0.32 (0.07)	0.11^***^	0.29 (0.07)	0.10^***^	−0.15 (0.07)	−0.06^*^	−0.22 (0.06)	−0.09^***^	0.09 (0.05)	0.04	0.11 (0.03)	0.08^**^	−0.09 (0.05)	−0.04	−0.01 (0.03)	−0.01
Support									0.08 (0.04)	0.10^*^	0.11 (0.02)	0.23^***^	−0.01 (0.03)	−0.01	0.05 (0.02)	0.11^*^
Collaboration									0.04 (0.04)	0.06	−0.03 (0.02)	−0.06	0.08 (0.03)	0.12^*^	0.03 (0.02)	0.07
Directing									0.03 (0.02)	0.03	−0.04 (0.01)	−0.07^**^	0.01 (0.02)	0.01	−0.02 (0.01)	−0.04
Noninvolvement									0.05 (0.03)	0.06	−0.09 (0.02)	−0.16^***^	0.01 (0.02)	0.01	−0.02 (0.02)	−0.04
Disengagement													0.06 (0.02)	0.06^**^	0.02 (0.01)	0.03
Engagement													0.54 (0.04)	0.37^***^	0.26 (0.02)	0.28^***^
**Indirect effects**																
Support													0.07 (0.01)	0.09^***^	0.03 (0.01)	0.07^***^
Collaboration													−0.01 (0.01)	−0.02	−0.01 (0.01)	−0.02
Directing													−0.02 (0.01)	0.03^**^	−0.01 (0.004)	−0.02^**^
Noninvolvement													−0.05 (0.01)	−0.06^***^	−0.02 (0.005)	−0.04^***^
**Total effects**																
Support													0.06 (0.04)	0.08	0.08 (0.02)	0.18^***^
Collaboration													0.07 (0.04)	0.10^*^	0.02 (0.02)	0.05
Directing													−0.01 (0.02)	−0.01	−0.03 (0.01)	−0.06^*^
Noninvolvement													−0.04 (0.02)	−0.05	−0.04 (0.02)	−0.08^**^

Unstandardized coefficient (standard error) standardized coefficient, presented. *n* = 1,489. *p < 0.05; **p < 0.01; ***p < 0.001.

**Table 4:** Direct, indirect, and total effects of shared and nonshared agency from fathers on occupational outcomes.

	Support		Collaboration		Directing		Noninvolvement		Disengagement		Engagement		Exploration		Satisfaction	
	*B(SE)*	*β*	*B(SE)*	*β*	*B(SE)*	*β*	*B(SE)*	*β*	*B(SE)*	*β*	*B(SE)*	*β*	*B(SE)*	*β*	*B(SE)*	*β*
**Direct effects**																
Age	−0.01 (0.01)	−0.02	−0.03 (0.01)	−0.07^**^	−0.06 (0.01)	−0.15^***^	0.04 (0.01)	0.10^***^	−0.01 (0.01)	−0.04	−0.01 (01)	−0.03	−0.01 (0.01)	−0.02	0.01 (0.01)	0.03
Gender (1 = Female)	0.07 (0.08)	0.02	−0.06 (0.08)	−0.02	−0.42 (0.07)	−0.16^***^	−0.02 (0.07)	−0.01	0.10 (0.06)	0.05	0.13 (0.04)	0.09^***^	−0.06 (0.05)	−0.03	0.02 (0.03)	0.01
Support									0.04 (0.04)	0.06	0.08 (0.02)	0.17^**^	0.01 (0.03)	0.01	0.002 (0.02)	0.01
Collaboration									0.06 (0.04)	0.09	0.01 (0.03)	0.03	0.08 (0.04)	0.11^*^	0.06 (0.02)	0.13^*^
Directing									0.003 (0.02)	0.003	−0.05 (0.01)	−0.10^***^	−0.03 (0.02)	−0.04	−0.03 (0.01)	−0.06^*^
Noninvolvement									0.04 (0.02)	0.05	−0.04 (0.02)	−0.07^*^	0.02 (0.02)	0.02	−0.03 (0.01)	−0.07^*^
Disengagement													0.07 (0.02)	0.07^**^	0.02 (0.02)	0.03
Engagement													0.55 (0.04)	0.37^***^	0.27 (0.02)	0.29^***^
**Indirect effects**																
Support													0.05 (0.01)	0.07^**^	0.02 (0.01)	0.05^**^
Collaboration													0.01 (0.01)	0.02	0.01 (0.01)	0.01
Directing													−0.03 (0.01)	−0.04^**^	−0.01 (0.004)	−0.03^**^
Noninvolvement													−0.02 (0.01)	−0.02	−0.01 (0.004)	−0.02
**Total effects**																
Support													0.05 (0.04)	0.08	0.02 (0.02)	0.06
Collaboration													0.09 (0.04)	0.13^*^	0.06 (0.02)	0.14^*^
Directing													−0.06 (0.02)	−0.07^**^	−0.05 (0.01)	−0.09^**^
Noninvolvement													0.00 (0.02)	0.00	−0.04 (0.02)	−0.09^**^

Unstandardized coefficient (standard error) standardized coefficient, presented. *n* = 1,489. *p < 0.05; **p < 0.01; ***p < 0.001.

**Table 5:** Direct, indirect, and total effects of shared and nonshared agency from VIP on occupational outcomes.

	Support		Collaboration		Directing		Noninvolvement		Disengagement		Engagement		Exploration		Satisfaction	
	*B(SE)*	*β*	*B(SE)*	*β*	*B(SE)*	*β*	*B(SE)*	*β*	*B(SE)*	*β*	*B(SE)*	*β*	*B(SE)*	*β*	*B(SE)*	*β*
**Direct effects**																
Age	0.03 (0.01)	0.08^**^	0.01 (0.01)	0.01	−0.05 (0.01)	−0.12^***^	0.005 (0.01)	0.01	−0.01 (0.01)	−0.04	−0.001 (0.01)	−0.005	0.0002 (0.01)	0.0006	0.01 (0.01)	0.05
Gender (1 = Female)	0.38 (0.08)	0.14^***^	0.28 (0.08)	0.10^***^	−0.25 (0.07)	−0.10^**^	−0.21 (0.07)	−0.09^**^	0.09 (0.06)	0.04	0.06 (0.04)	0.04	−0.06 (0.06)	−0.03	0.02 (0.04)	0.01
Support									−0.06 (0.04)	−0.07	0.10 (0.02)	0.19^***^	0.07 (0.04)	0.09	0.04 (0.02)	0.07
Collaboration									0.10 (0.04)	0.13^*^	0.03 (0.03)	0.07	0.01 (0.04)	0.02	0.01 (0.02)	0.01
Directing									0.05 (0.03)	0.06	−0.06 (0.02)	−0.10^**^	0.01 (0.02)	0.01	0.02 (0.02)	0.03
Noninvolvement									0.04 (0.03)	0.04	−0.08 (0.02)	−0.13^***^	−0.02 (0.03)	−0.02	−0.03 (0.02)	−0.05
Disengagement													0.06 (0.03)	0.06^*^	0.03 (0.02)	0.04
Engagement													0.50 (0.04)	0.34^***^	0.26 (0.03)	0.28^***^
**Indirect effects**																
Support													0.05 (0.01)	0.06^**^	0.02 (0.01)	0.05^**^
Collaboration													0.02 (0.01)	0.03	0.01 (0.01)	0.02
Directing													−0.02 (0.01)	−0.03^**^	−0.01 (0.005)	−0.03^**^
Noninvolvement													−0.04 (0.01)	−0.04^***^	−0.02 (0.01)	−0.03^***^
**Total effects**																
Support													0.11 (0.04)	0.15^**^	0.06 (0.02)	0.12^*^
Collaboration													0.03 (0.04)	0.05	0.02 (0.02)	0.04
Directing													−0.01 (0.02)	−0.02	0.003 (0.02)	0.01
Noninvolvement													−0.06 (0.03)	−0.06^*^	−0.05 (0.02)	−0.09^**^

Unstandardized coefficient (standard error) standardized coefficient, presented. *n* = 1,256. *p < 0.05; **p < 0.01; ***p < 0.001.

**Table 6:** Direct, indirect, and total effects of shared and nonshared agency from romantic partners on occupational outcomes.

	Support		Collaboration		Directing		Noninvolvement		Disengagement		Engagement		Exploration		Satisfaction	
	*B(SE)*	*β*	*B(SE)*	*β*	*B(SE)*	*β*	*B(SE)*	*β*	*B(SE)*	*β*	*B(SE)*	*β*	*B(SE)*	*β*	*B(SE)*	*β*
**Direct effects**																
Age	0.10 (0.01)	0.25^***^	0.08 (0.01)	0.18^***^	−0.0002 (0.01)	−0.0005	−0.01 (0.01)	−0.02	−0.02 (0.01)	−0.06	−0.01 (0.01)	−0.03	−0.00 (0.01)	−0.01	0.004 (0.01)	0.02
Gender (1 = Female)	0.49 (0.10)	0.17^***^	0.36 (0.10)	0.12^***^	−0.31 (0.09)	−0.12^**^	−0.11 (0.09)	−0.05	−0.004 (0.08)	−0.002	0.06 (0.05)	0.04	−0.10 (0.07)	−0.04	−0.07 (0.05)	−0.05
Support									−0.05 (0.05)	−0.07	0.13 (0.03)	0.26^***^	0.003 (0.04)	0.004	−0.02 (0.03)	−0.04
Collaboration									0.14 (0.05)	0.19^**^	−0.01 (0.03)	−0.01	0.11 (0.04)	0.15^*^	0.07 (0.03)	0.16^*^
Directing									−0.0007 (0.03)	−0.0008	−0.06 (0.02)	−0.10^*^	−0.05 (0.03)	−0.06	−0.02 (0.02)	−0.05
Noninvolvement									0.01 (0.03)	0.01	−0.09 (0.02)	−0.15^***^	−0.01 (0.03)	−0.01	−0.02 (0.02)	−0.03
Disengagement													0.05 (0.03)	0.05	0.04 (0.02)	0.07^*^
Engagement													0.47 (0.05)	0.33^***^	0.25 (0.03)	0.28^***^
**Indirect effects**																
Support													0.06 (0.02)	0.08^***^	0.03 (0.01)	0.07^**^
Collaboration													0.003 (0.02)	0.01	0.005 (0.01)	0.01
Directing													−0.03 (0.01)	−0.03^*^	−0.01 (0.01)	−0.03^*^
Noninvolvement													−0.04 (0.01)	−0.05^***^	−0.02 (0.01)	−0.04^***^
**Total effects**																
Support													0.06 (0.05)	0.09	0.01 (0.03)	0.03
Collaboration													0.12 (0.05)	0.16^*^	0.08 (0.03)	0.17^*^
Directing													−0.08 (0.03)	−0.10^*^	−0.04 (0.02)	−0.07
Noninvolvement													−0.06 (0.03)	−0.06	−0.04 (0.02)	−0.07

Unstandardized coefficient (standard error) standardized coefficient, presented. *n* = 815. *p < 0.05; **p < 0.01; ***p < 0.001.

### Shared and nonshared agency with mother

Results of the model using shared and nonshared agency with mother are reported in Table 3. As can be seen, age and gender differences were significant (see rows 1 and 2). Mothers were perceived to be less involved in the career development of an older compared to a younger child (i.e., age was negatively related to support, collaboration and directing, and positively related to noninvolvement). Daughters compared to sons were more likely to perceive shared agency with mothers (i.e., support and collaboration) and less likely to perceive nonshared agency with mothers (directing and noninvolvement).

The direct effects of maternal shared and nonshared agency on goal engagement/disengagement and occupational outcomes are displayed in Table 3. Shared agency with mothers (rows 3 and 4) was generally beneficial whereas nonshared agency (rows 5 and 6) generally had a negative association with young adult’s career outcomes. Although it should be noted that collaboration (row 4) was not significantly related to individual motivation (i.e., disengagement and engagement), perceived maternal support was positively related to career goal engagement. Unexpectedly, maternal support was positively related to goal disengagement. It can also be seen that nonshared agency strategies (i.e., directing and noninvolvement) were not significantly related to career goal disengagement as expected, but were negatively and significantly related to goal engagement.

For the relations between shared agency and occupational outcomes, we found that support was positively linked with occupational satisfaction (row 13) mostly *via* an indirect path through motivational strategies (row 9). However, one can see that collaboration was positively linked with exploration (row 14) mostly independent of motivational strategies (i.e., there was no significant indirect effect, see row 10). For nonshared agency, directing and noninvolvement both had negative relationships with occupational satisfaction (rows 15–16) *via* motivational strategies (see rows 11 and 12).

### Shared and nonshared agency with father

Table 4 details the results of the model that uses shared and nonshared agency with fathers. As can be seen, a similar but less consistent pattern was found between age and shared/nonshared agency with fathers. Older participants reported less collaboration with or direction from their fathers and were more likely to report paternal noninvolvement than younger participants (see row 1). Gender differences were also found with sons more likely to report that their fathers directed their occupational pursuits than daughters (row 2).

Consistent with findings relating to mothers, perceived support was positively related to goal engagement (row 3), whereas both directing and noninvolvement were negatively related to goal engagement (rows 5–6).

Turning to the relations between shared agency and occupational outcomes, we found that perceived collaboration was positively linked to both occupational exploration and satisfaction (row 4), independent of motivational strategies (see significant association in row 14 but not in row 10). We also found that perceived support was positively linked with both exploration and satisfaction *via* indirect pathways through motivational strategies (row 9). Directing was negatively related to exploration and satisfaction mostly *via* motivational strategies of goal engagement and disengagement (row 11), while noninvolvement was negatively related to satisfaction independent of motivational strategies (see significant association on row 16 but not in row 12).

### Shared and nonshared agency with VIP

Table 5 includes results relating to shared and nonshared agency with important nonparental adults (VIPs). In contrast to parental shared and nonshared agency, we found that older participants reported more support from VIPs (row 1). However, similar to parental shared and nonshared agency, we also found that older participants reported less directing from VIPs than younger participants (also row 1). Female participants reported greater VIP support and collaboration and less VIP directing and noninvolvement than male participants (row 2).

Regarding links between shared and nonshared agency and motivational strategies, we found that support was positively related with goal engagement (row 3), while directing and noninvolvement were negatively related with engagement (rows 5 and 6). Contrary to our hypotheses, perceived collaboration with a VIP was positively related to career goal disengagement (row 4).

For the occupational outcomes under study, we found that support was positively related to occupational exploration and occupational satisfaction *via* motivational strategies (rows 9 and 13). In contrast, noninvolvement was negatively linked to occupational exploration and satisfaction (row 16) *via* motivational strategies (row 12). There was also a negative indirect path from directing to occupational exploration and satisfaction *via* motivational strategies (row 11). Collaboration with VIPs, however, was not significantly related to occupational outcomes.

### Shared and nonshared agency with romantic partner

Table 6 reports the results of the model investigating shared and nonshared agency with one’s romantic partner. We found that older participants were more likely to report shared agency (support and collaboration) with their romantic partners than younger participants (row 1). Female participants were also more likely to report support and collaboration and less likely to report directing from their romantic partners than male participants (row 2).

Similar to results with other relationship partners, support was positively related to career goal engagement, while directing and noninvolvement were negatively related to career goal engagement. We also found that collaboration was positively related to career goal disengagement, which was similar to collaboration with VIPs (row 4).

As for the relations between shared and nonshared agency with occupational outcomes, we found that collaboration was positively related to occupational exploration and occupational satisfaction independent of motivational strategies (significant associations on row 14 but not on row 10). Support was also positively related to occupational exploration and satisfaction, but only *via* an indirect pathway through motivational strategies (row 9) and the total effects of support on occupational exploration and satisfaction were nonsignificant (row 13). Consistent with other social partners, directing was negatively related to occupational exploration, mostly *via* motivational strategies (row 15). Directing was also negatively related to occupational satisfaction *via* motivational strategies (row 11), but the total effect of directing on occupational satisfaction was nonsignificant (row 15). Similarly, noninvolvement was negatively related to both occupational exploration and occupational satisfaction *via* indirect pathways through motivational strategies (row 12), but the total effect of noninvolvement on these occupational outcomes was nonsignificant (row 16).

### Shared and nonshared agency with all relationship partners

A supplementary analysis was conducted including shared and nonshared agency from all relationship partners in the same model, controlling for age and gender. Because of the number of pathways in this model, only the significant total, indirect, and direct effects are presented here.

Results indicate that when patterns of shared and nonshared agency with each relationship partner were included in the same model, collaboration with romantic partner was the only significant predictor of career goal disengagement (*β* = 0.21, *p* = 0.027). Support from mothers was the only positive predictor (*β* = 0.23, *p* = 0.010), while collaboration with mothers (*β* = −0.31, *p* = 0.002) and directing from mothers (*β* = −0.24, *p* = 0.001) were the only negative predictors of career goal engagement. Support from mothers was positively related to occupational satisfaction (*β* = 0.21, *p* = 0.023), *via* motivational strategies (nonsignificant direct effect, significant indirect effect). While the total effects were nonsignificant, there were significant indirect effects of support from mothers on occupational exploration *via* motivational strategies (*β* = 0.08, *p* = 0.010), of collaboration from mothers on occupational exploration (*β* = −0.10, *p* = 0.003) and satisfaction (*β* = −0.08, *p* = 0.006), and of noninvolvement from mothers on occupational exploration (*β* = −0.07, *p* = 0.003) and satisfaction (*β* = −0.06, *p* = 0.003). Directing from romantic partners negatively predicted both occupational exploration (*β* = −0.15, *p* = 0.009) and satisfaction (*β* = −0.14, *p* = 0.016), independently of motivational strategies (significant direct effects, nonsignificant indirect effects). Finally, in contrast to collaboration from mothers, collaboration from fathers was positively related to occupational satisfaction (*β* = 0.23, *p* = 0.041); however, neither the direct nor indirect effects were significant.

## Discussion

Young adults’ pursuit of occupational goals can be helped or hindered by their social partners. However, to our knowledge, current theory and assessments do not capture the *joint* pursuit of occupational goals with important others in one’s social network (e.g., collaboration) and/or only consider the parent–child dyad (e.g., Keller and Whiston, 2008; Sawitri and Creed, 2021). This study provides a starting point by illustrating that shared agency is generally positively associated with career development, while nonshared agency is generally negatively associated with career development. These effects were most consistent for support and noninvolvement, wherein the relationship partner was more passive. When the relationship partner took on a more active role, as in the case of collaboration and directing, the associations with career development were more complex and likely to vary across relationships. Although our study design was cross-sectional and self-report, the main contributions to the literature are twofold. First, the identification of occupational shared and nonshared agency patterns provides a framework to understand how social partners impede or facilitate career development. Second, the inclusion of individual motivation as a potential mediator of shared and nonshared agency offers a possible mechanism through which social relationships can influence career development.

The transition from school-to-work enables an unprecedented variety of ways for young adults to communicate and interact with their parents as they launch into careers and other adult roles. We found that while parental support remained relatively stable across ages, at older ages of young adulthood, parents were more likely to become uninvolved with their child’s career development, and young adults were less likely to report that they collaborated with or were directed by their parents in their career pursuits. Similarly, Giordano et al. (2008) found that young adulthood was characterized by reduced parental influence, which was somewhat replaced by the influence and support of romantic partners. Gender differences in parental experiences in work and in family life appear to facilitate gender-matched opportunities for shared agency. Daughters were more likely to report shared agency with their mothers and to feel like their fathers directed their career goal pursuits, while sons were more likely to report nonshared agency with their mothers. This is consistent with research on parent gender and childrearing styles such that fathers are typically regarded as more authoritarian than mothers (i.e., more directing and demanding obedience), while mothers are perceived as warmer and more nurturant than fathers (i.e., more supportive; Smetana, 1995). While preliminary, our findings further suggest that shared and nonshared agency may be differently associated with career development depending upon parent gender (Nota et al., 2012).

By differentiating the shared agency subtypes and by including individual motivational variables, we accomplished two goals. First, we showed that social support is positively associated with developmental outcomes *via* individual motivational engagement and disengagement strategies (see also Heckhausen et al., 2010; Kriegbaum et al., 2016). We also show that joint goal pursuit, i.e., collaboration, is generally promotive of development (Young et al., 2001). Parents may feel that more is at stake when older children are closer to career entry and become more involved—and thus support and collaborate with their young adult children. Similarly, young adults may turn to their parents for help as they navigate the complexities of the school-to-work transition. However, our findings of negative associations between patterns of nonshared agency (directing, noninvolvement) and career development suggest that unidirectional relational dynamics are not adaptive. Finally, our analyses suggest that collaboration may be more welcome, while directing may be more detrimental when experienced with fathers than with mothers, but these tentative findings would need more research to unpack.

In contrast to parents, important non-parental adults (VIPs) and romantic partners are typically actively recruited into one’s social network. Our results generally show similarities in function between these two types of chosen social partners. Perceived support from both VIPs and romantic partners can promote career development, while noninvolvement can hinder career development. However, only collaboration with romantic partners was beneficial for career development. Romantic partners are an understudied relationship in early career development, yet our results suggest that they play an important role in jointly guiding an individual’s career development.

### Limitations and future directions

There are several methodological limitations that are worthy to note. As mentioned earlier, our study’s cross-sectional design does not allow us to examine the processes involved as they unfold over time, and which direction of influence is significant and potentially dominant. It is just as likely that individuals who are highly goal engaged can elicit shared agency from others. Similarly, those who are highly goal disengaged can elicit others’ noninvolvement or directing instead of the other way around. Additionally, the use of self-report only captures perceptions of behaviors and intentions, which further limit the scope of our findings to subjective experiences.

Our sample selection has both advantages and disadvantages. An advantage is the multiple sites we used for data collection, including young adults recruited through Amazon’s Mechanical Turk. Participants recruited *via* Mechanical Turk were more likely to be older, White, male, and have a lower subjective SES. Attesting to these demographic differences, participants recruited *via* Mechanical Turk also reported generally lower levels of parental involvement and higher levels of non-parental involvement in their occupational shared agency scale responses. While the full sample was demographically diverse in several ways, it is still limited in its generalizability, particularly to low-income young adults, young adults without postsecondary education experience, and those living outside of the U.S. For example, nonshared agency (directing) may have less negative outcomes for some individuals including those who are less engaged or for young adults whose culture normalizes intense and directive parenting. Future research should examine cross-cultural similarities and differences in occupational shared agency to better inform culturally specific practices.

Finally, although our overall pattern of results supports our hypotheses, not all findings were fully consistent with our expectations. Unexpected findings, such as negative aspects of shared agency or positive aspects of nonshared agency, should be interpreted with caution and warrant further research. Finally, our research was focused on relationship dyads, and our measure was unidirectional. Future research that expands the concept of shared agency to multiple-partner units and assesses shared agency perceptions from all partners would allow better insights into the interdependence of occupational shared and nonshared agency.

### Conclusion and implications

Prior research has underscored the importance of social partners for career development, particularly during young adulthood. The present study contributes to the career development field by showing how one’s own occupational engagement and the perceived shared agency from close relationship partners are essential for positive career development during young adulthood. While our findings are specific to young adults, shared and nonshared agency with important social relationships likely continue to play an important role in career development during later ages and career stages.

Our findings also have practical implications. Perhaps the most relevant implications are for young adults in settings which offer extensive career counseling services, such as universities. However, young adults not enrolled in college may access external career counselors or similar supportive services *via* therapy/counseling or reemployment programs. In general, career counselors should consider the ways in which young adults and their close social relationship partners coregulate career development in unique and bidirectional ways, and how these changes across goal phases. When young adults are not focused on career, they may benefit most from the active and directing influence of close relationship partners. When young adults are actively engaged in goal pursuit, they may benefit most from passive yet supportive relationships. Moreover, career counselors should consider an individual’s age and developmental period to help identify the relative influence of different social relationships on their career development. Our results suggest that young adults are more receptive to collaborative patterns of shared agency with their romantic partner than with their parents. This shift toward nonparental relationships, such as peers, mentors, and romantic partners should become more pronounced as adolescents transition into adulthood. Finally, career counselors should consider the ways in which major social relationships promote or impair an individual’s career development through their actions and inactions. By taking a more comprehensive assessment of shared and nonshared agency patterns across an individual’s multiple close social relationships, specific interventions can help promote the positive coregulation of career development in a young adult’s close social network.

## Data availability statement

The raw data supporting the conclusions of this article will be made available by the authors, without undue reservation.

## Ethics statement

The studies involving human participants were reviewed and approved by UCI and Soka University of America. Written informed consent was not provided because this was an online study. We gave all participants study information before giving them the option to start the study in lieu of signed informed consent.

## Author contributions

EC, JS, BV, and JH contributed to conception and design of the study. JS organized the database, performed the statistical analysis, and wrote sections of the manuscript. EC wrote the first draft of the manuscript. All authors contributed to the article and approved the submitted version.

## Conflict of interest

The authors declare that the research was conducted in the absence of any commercial or financial relationships that could be construed as a potential conflict of interest.

## Publisher’s note

All claims expressed in this article are solely those of the authors and do not necessarily represent those of their affiliated organizations, or those of the publisher, the editors and the reviewers. Any product that may be evaluated in this article, or claim that may be made by its manufacturer, is not guaranteed or endorsed by the publisher.

## Footnotes

^1^Compared to participants recruited through a University, participants recruited *via* Mechanical Turk were more likely to be older [*M* = 26.10 vs. 20.13; *t*(1,745) = 41.56, *p* < 0.001], identify as male [61% vs. 28%; *t*(1721) = 11.24, *p* < 0.001], and identify as White [72% vs. 25%; *t*(1,741) = 16.70, *p* < 0.001], and less likely to identify as Black/African American [6% vs. 30%; *t*(1,741) = −8.92, *p* < 0.001] or Hispanic/Latinx White [4% vs. 22%; *t*(1,741) = −7.32, *p* < 0.001], and reported a lower subjective SES [*M* = 5.21 vs. 4.53; *t*(1,661) = −5.42, *p* < 0.001].
