# Supplementary material for: Shared and nonshared agency for occupational goals with mothers, fathers, VIPs, and romantic partners during young adulthood
Source: Front Psychol. 2022 Nov 18;13:902288. doi: 10.3389/fpsyg.2022.902288 (PMC9716139; doi:10.3389/fpsyg.2022.902288)
Supplement: Supplementary file 1 [file Data_Sheet_1.PDF]

*** Syntax for mediation models**

***shared and nonshared agency with mothers**

```
sem (age female -> mom_supportm mom_collabm mom_directm mom_noninvm) ///
(mom_supportm mom_collabm mom_directm mom_noninvm age female -> ops_dis ///
ops_engagement) (mom_supportm mom_collabm mom_directm mom_noninvm ops_dis ///
ops_engagement age female -> occ_plan occ_sat), ///
cov(e.mom_supportm*e.mom_collabm e.mom_supportm*e.mom_directm ///
e.mom_supportm*e.mom_noninvm e.mom_collabm*e.mom_directm ///
e.mom_collabm*e.mom_noninvm e.mom_directm*e.mom_noninvm ///
e.ops_dis*e.ops_engagement e.occ_plan*e.occ_sat age*female) nolog stand
estat teffects, stand
```

***shared and nonshared agency with fathers**

```
sem (age female -> dad_supportm dad_collabm dad_directm dad_noninvm) ///
(dad_supportm dad_collabm dad_directm dad_noninvm age female -> ops_dis ///
ops_engagement) (dad_supportm dad_collabm dad_directm dad_noninvm ops_dis ///
ops_engagement age female -> occ_plan occ_sat), ///
cov(e.dad_supportm*e.dad_collabm e.dad_supportm*e.dad_directm ///
e.dad_supportm*e.dad_noninvm e.dad_collabm*e.dad_directm ///
e.dad_collabm*e.dad_noninvm e.dad_directm*e.dad_noninvm ///
e.ops_dis*e.ops_engagement e.occ_plan*e.occ_sat age*female) nolog stand
estat teffects, stand
```

***shared and nonshared agency with VIPs**

```
sem (age female -> vip_supportm vip_collabm vip_directm vip_noninvm) ///
(vip_supportm vip_collabm vip_directm vip_noninvm age female -> ops_dis ///
ops_engagement) (vip_supportm vip_collabm vip_directm vip_noninvm ///
ops_dis ops_engagement age female -> occ_plan occ_sat), ///
cov(e.vip_supportm*e.vip_collabm e.vip_supportm*e.vip_directm ///
e.vip_supportm*e.vip_noninvm e.vip_collabm*e.vip_directm ///
e.vip_collabm*e.vip_noninvm e.vip_directm*e.vip_noninvm ///
e.ops_dis*e.ops_engagement e.occ_plan*e.occ_sat age*female) nolog stand
estat teffects, stand
```

***shared and nonshared agency with romantic partners**

```
sem (age female -> so_supportm so_collabm so_directm so_noninvm) ///
(so_supportm so_collabm so_directm so_noninvm age female -> ops_dis ///
ops_engagement) (so_supportm so_collabm so_directm so_noninvm ops_dis ///
ops_engagement age female -> occ_plan occ_sat), ///
cov(e.so_supportm*e.so_collabm e.so_supportm*e.so_directm ///
e.so_supportm*e.so_noninvm e.so_collabm*e.so_directm ///
e.so_collabm*e.so_noninvm e.so_directm*e.so_noninvm ///
e.ops_dis*e.ops_engagement e.occ_plan*e.occ_sat age*female) nolog
estat teffects, stand
```

***shared and nonshared agency with all 4 relationship partners**

```
sem (age female -> mom_supportm mom_collabm mom_directm mom_noninvm ///
dad_supportm dad_collabm dad_directm dad_noninvm vip_supportm vip_collabm ///
vip_directm vip_noninvm so_supportm so_collabm so_directm so_noninvm) ///
(mom_supportm mom_collabm mom_directm mom_noninvm dad_supportm dad_collabm ///
```

```
dad_directm dad_noninvm vip_supportm vip_collabm vip_directm vip_noninvm ///
so_supportm so_collabm so_directm so_noninvm age female -> ops_cscd ///
ops_engagement) (mom_supportm mom_collabm mom_directm mom_noninvm ///
dad_supportm dad_collabm dad_directm dad_noninvm vip_supportm vip_collabm ///
vip_directm vip_noninvm so_supportm so_collabm so_directm so_noninvm ///
ops_cscd ops_engagement age female -> occ_plan occ_sat), ///
cov(e.mom_supportm*e.mom_collabm e.mom_supportm*e.mom_directm ///
e.mom_supportm*e.mom_noninvm e.mom_collabm*e.mom_directm ///
e.mom_collabm*e.mom_noninvm e.mom_directm*e.mom_noninvm ///
e.dad_supportm*e.dad_collabm e.dad_supportm*e.dad_directm ///
e.dad_supportm*e.dad_noninvm e.dad_collabm*e.dad_directm ///
e.dad_collabm*e.dad_noninvm e.dad_directm*e.dad_noninvm ///
e.vip_supportm*e.vip_collabm e.vip_supportm*e.vip_directm ///
e.vip_supportm*e.vip_noninvm e.vip_collabm*e.vip_directm ///
e.vip_collabm*e.vip_noninvm e.vip_directm*e.vip_noninvm ///
e.so_supportm*e.so_collabm e.so_supportm*e.so_directm ///
e.so_supportm*e.so_noninvm e.so_collabm*e.so_directm ///
e.so_collabm*e.so_noninvm e.so_directm*e.so_noninvm ///
e.mom_supportm*e.dad_supportm e.mom_supportm*e.dad_collabm ///
e.mom_supportm*e.dad_directm e.mom_supportm*e.dad_noninvm ///
e.mom_supportm*e.vip_supportm e.mom_supportm*e.vip_collabm ///
e.mom_supportm*e.vip_directm e.mom_supportm*e.vip_noninvm ///
e.mom_supportm*e.so_supportm e.mom_supportm*e.so_collabm ///
e.mom_supportm*e.so_directm e.mom_supportm*e.so_noninvm ///
e.mom_collabm*e.dad_supportm e.mom_collabm*e.dad_collabm ///
e.mom_collabm*e.dad_directm e.mom_collabm*e.dad_noninvm ///
e.mom_collabm*e.vip_supportm e.mom_collabm*e.vip_collabm ///
e.mom_collabm*e.vip_directm e.mom_collabm*e.vip_noninvm ///
e.mom_collabm*e.so_supportm e.mom_collabm*e.so_collabm ///
e.mom_collabm*e.so_directm e.mom_collabm*e.so_noninvm ///
e.mom_directm*e.dad_supportm e.mom_directm*e.dad_collabm ///
e.mom_directm*e.dad_directm e.mom_directm*e.dad_noninvm ///
e.mom_directm*e.vip_supportm e.mom_directm*e.vip_collabm ///
e.mom_directm*e.vip_directm e.mom_directm*e.vip_noninvm ///
e.mom_directm*e.so_supportm e.mom_directm*e.so_collabm ///
e.mom_directm*e.so_directm e.mom_directm*e.so_noninvm ///
e.mom_noninvm*e.dad_supportm e.mom_noninvm*e.dad_collabm ///
e.mom_noninvm*e.dad_directm e.mom_noninvm*e.dad_noninvm ///
e.mom_noninvm*e.vip_supportm e.mom_noninvm*e.vip_collabm ///
e.mom_noninvm*e.vip_directm e.mom_noninvm*e.vip_noninvm ///
e.mom_noninvm*e.so_supportm e.mom_noninvm*e.so_collabm ///
e.mom_noninvm*e.so_directm e.mom_noninvm*e.so_noninvm ///
e.dad_supportm*e.vip_supportm e.dad_supportm*e.vip_collabm ///
e.dad_supportm*e.vip_directm e.dad_supportm*e.vip_noninvm ///
e.dad_supportm*e.so_supportm e.dad_supportm*e.so_collabm ///
e.dad_supportm*e.so_directm e.dad_supportm*e.so_noninvm ///
e.dad_collabm*e.vip_supportm e.dad_collabm*e.vip_collabm ///
e.dad_collabm*e.vip_directm e.dad_collabm*e.vip_noninvm ///
e.dad_collabm*e.so_supportm e.dad_collabm*e.so_collabm ///
e.dad_collabm*e.so_directm e.dad_collabm*e.so_noninvm ///
e.dad_directm*e.vip_supportm e.dad_directm*e.vip_collabm ///
```

```
e.dad_directm*e.vip_directm e.dad_directm*e.vip_noninvm ///
e.dad_directm*e.so_supportm e.dad_directm*e.so_collabm ///
e.dad_directm*e.so_directm e.dad_directm*e.so_noninvm ///
e.dad_noninvm*e.vip_supportm e.dad_noninvm*e.vip_collabm ///
e.dad_noninvm*e.vip_directm e.dad_noninvm*e.vip_noninvm ///
e.dad_noninvm*e.so_supportm e.dad_noninvm*e.so_collabm ///
e.dad_noninvm*e.so_directm e.dad_noninvm*e.so_noninvm ///
e.vip_supportm*e.so_supportm e.vip_supportm*e.so_collabm ///
e.vip_supportm*e.so_directm e.vip_supportm*e.so_noninvm ///
e.vip_collabm*e.so_supportm e.vip_collabm*e.so_collabm ///
e.vip_collabm*e.so_directm e.vip_collabm*e.so_noninvm ///
e.vip_directm*e.so_supportm e.vip_directm*e.so_collabm ///
e.vip_directm*e.so_directm e.vip_directm*e.so_noninvm ///
e.vip_noninvm*e.so_supportm e.vip_noninvm*e.so_collabm ///
e.vip_noninvm*e.so_directm e.vip_noninvm*e.so_noninvm ///
e.ops_cscd*e.ops_engagement e.occ_plan*e.occ_sat age*female) nolog stand
estat teffects, stand
```
